# Distribution of ST116 carbapenem-resistant *Citrobacter freundii* in public genomes and characterization of a triple-carbapenemase-producing strain

**DOI:** 10.3389/fcimb.2026.1846731

**Published:** 2026-07-01

**Authors:** Wanxiang Li, Jie Ma, Cong Zhang, Yuhui Wang, Yang Duan

**Affiliations:** Department of Clinical Laboratory, Weifang People’s Hospital, Shandong Second Medical University, Weifang, China

**Keywords:** carbapenem resistance, *Citrobacter freundii*, genomic epidemiology, hybrid plasmid, ST116

## Abstract

**Objective:**

To characterize a carbapenem-resistant *Citrobacter freundii* (CR-CFR) clinical isolate belonging to the sequence type ST116 and to investigate the public-genome distribution of ST116 among carbapenem-resistant *C. freundii*.

**Methods:**

A carbapenem-resistant *C. freundii* isolate (WF1033) was recovered from a patient with a urinary tract infection. Antimicrobial susceptibility testing, whole-genome sequencing, and detailed plasmid structural analyses were performed. To contextualize this isolate, a large-scale genomic analysis of publicly available CR-CFR genomes was conducted, including multilocus sequence typing (MLST), minimum spanning tree analysis, global distribution mapping, and phylogenetic reconstruction of sequence type ST116.

**Results:**

WF1033 exhibited an extensively drug-resistant phenotype and harbored three carbapenemase genes, *bla*_KPC-2_, *bla*_IMP-8_, and *bla*_NDM-1_. Genomic analysis revealed that WF1033 belongs to ST116 and carries four plasmids. Notably, *bla*_KPC-2_ and *bla*_IMP-8_ were co-located on a novel hybrid IncFIB/IncFII plasmid (pWF1033-KPC-IMP), formed through the integration of distinct mobile genetic elements, whereas *bla*_NDM-1_ was carried on a typical IncX3 plasmid. Analysis of 2,436 CR-CFR genomes identified 171 sequence types, with ST22 being the most prevalent globally. However, ST116 showed marked regional enrichment in China, accounting for 56.97% of all ST116 isolates. ST116 also displayed a higher proportion of strains carrying multiple carbapenem resistance genes (CRGs) compared with other major sequence types. Phylogenetic analysis of 165 ST116 isolates revealed multiple clades, suggesting possible lineage expansion and geographic persistence within this sequence type, with WF1033 representing the only ST116 strain identified to date carrying three CRGs simultaneously.

**Conclusions:**

Our findings suggest that ST116 represents a lineage associated with increased carriage of multiple carbapenem resistance genes in CR-CFR. Continuous genomic surveillance is warranted to monitor the dissemination of multidrug-resistant *C. freundii*, particularly among ST116-associated lineages.

## Introduction

During recent decades, carbapenem resistance in Gram-negative bacteria has become a significant threat to global public health. The World Health Organization has recognized carbapenem-resistant *Enterobacterales* (CRE) as a critical group priority pathogen (Zhang et al., 2023b). The main resistance mechanism of CRE strains involves the production of carbapenemases, including *Klebsiella pneumoniae* carbapenemase (KPC), New Delhi metallo-beta-lactamase (NDM), and oxacillinase (OXA)-48-like carbapenemase. These enzymes can confer high levels of resistance to β-lactam antibiotic drugs commonly used in clinical practice (Nobrega et al., 2023). The carbapenem resistance genes (CRGs) that encode these enzymes in bacteria are mainly horizontally transmitted via mobile genetic elements (e.g., plasmids, transposons, and integrons), leading to a wide distribution of CRE strains around the world and increased isolation rates in recent years (Li et al., 2022; Nobrega et al., 2023).

*Citrobacter* spp., particularly *C. freundii*, are increasingly implicated in nosocomial infections (Li et al., 2022). A Gram-negative bacterium, *C. freundii* is related to various systemic infections, including urinary tract infections, pulmonary infections, meningitis, and bloodstream infections. Recent studies reported increasing isolation of *C. freundii* in clinical infection cases, with the phenotypic susceptibility of isolates indicating that its resistance rates were also increasing, particularly among carbapenemase-producing *Citrobacter* spp (Nobrega et al., 2023). Even more concerning is the continuous emergence of *Citrobacter* strains carrying two or even three CRGs in recent years. These strains make clinical treatment more difficult and pose a greater potential threat to public health (Wu et al., 2016; Han et al., 2022; Li et al., 2022; Nobrega et al., 2023).

This study analyzes the genomic characteristics of a ST116 CR-CFR strain that carries CRGs. An analysis was performed on ST116 strains among CR-CFR isolates worldwide. The molecular characteristics of this bacterium elucidate its drug resistance mechanisms and provide data to support understanding of the prevalence of ST116 CR-CFR.

## Materials and methods

### Clinical data and strain collection

Clinical data were sourced from an electronic health record platform. The carbapenem-resistant *C. freundii* (CR-CFR) strain WF1033 was isolated in December 2016 from a large tertiary teaching hospital in northern China. Identification was performed using Vitek-MS (bioMérieux, Marcy l’Etoile, France), with *Escherichia coli* (ATCC 8739) serving as the quality control strain.

### Antimicrobial susceptibility testing

The antimicrobial susceptibility of WF1033 was assessed using the BD Phoenix™ M50 system (Becton, Dickinson and Company, Franklin Lakes, NJ, USA) and the Kirby–Bauer disk diffusion method (Oxoid, Hampshire, UK), following the 2021 guidelines of the Clinical and Laboratory Standards Institute (CLSI). This evaluation included 22 commonly employed antimicrobial agents, including β-lactams (penicillins, carbapenems, cephalosporins), fluoroquinolones, aminoglycosides, and sulfonamide combinations. *Escherichia coli* ATCC 25922 was utilized for quality control purposes.

### Whole-genome sequencing, *de novo* assembly, and annotation

Genomic DNA of WF1033 was extracted using an Omega Bio-Tek kit (Norcross, GA, USA). Whole-genome sequencing was performed on an Illumina NovaSeq 6000 platform (San Diego, CA, USA). Quality control and data filtering were conducted using TrimGalore v0.5.0 (https://github.com/FelixKrueger/TrimGalore) and FastQC (https://www.bioinformatics.babraham.ac.uk/projects/fastqc/, accessed July 26, 2022). Additionally, WF1033 underwent long-read sequencing on the Nanopore PromethION platform (Oxford Nanopore Technologies, UK). Hybrid assembly of Illumina and Nanopore reads was performed using Unicycler v0.4.9 (Wick et al., 2017). For sequence annotation and comparison, open reading frames (ORFs) were predicted with RAST v2.0 and the RefSeq database, while BLASTN facilitated multiple and pairwise sequence comparisons. Gene structure figures were created using the DANMEL database (Wang et al., 2022).

### MLST and phylogenetic analysis of ST116 CR-CFR

Multilocus sequence typing (MLST) was performed to determine the STs of the isolates. Seven housekeeping genes (*aspC*, *clpX*, *fadD*, *mdh*, *arcA*, *dnaG*, *lysP*) were compared with the MLST database (https://pubmlst.org/). Global *C. freundii* genome sequences were downloaded from the NCBI database (up to May 28, 2025). Antimicrobial resistance genes (ARGs) were identified using ABRicate v1.0.1 (https://github.com/tseemann/abricate) with the CARD database ((https://card.mcmaster.ca/), and 2,436 *C. freundii* isolates carrying carbapenemase genes were defined as CR-CFR and included in the public-genome distribution analysis. All assemblies were confirmed as *C. freundii* by ANI analyses, with a threshold of >95% ANI. The minimum spanning tree-like structures were illustrated by PHYLOViZ software (Nascimento et al., 2017) via goeBURST Full MST based on allelic distances. Global map of CR-CFR strain visualized by CNSknowall (https://cnsknowall.com/#/PersonalCenter). These 165 ST116 strains were used to build the phylogenetic tree based on single-nucleotide polymorphisms (SNPs) by Mummer 3.25 (Delcher et al., 2003). MEGAX 10.1.8 was used to generate unrooted maximum-likelihood phylogenetic trees with a bootstrap iteration of 1000 (Kumar et al., 2018). The phylogenetic tree was visualized by iTOL (Letunic and Bork, 2019).

### Statistical analysis

Data were analyzed using *GraphPad Prism* 9 software. Count data were presented as numbers (n) and percentages (%). Pairwise Pearson chi-square tests were performed with ST116 as the reference group to compare with ST22 and other ST types. The test conditions were satisfied (total sample size > 40, expected frequencies all > 5). A two-tailed significance level of α = 0.05 was adopted; *P* < 0.05 indicated a statistically significant difference.

## Results

### Clinical characteristics and antimicrobial susceptibility of *C. freundii* WF1033

An 85-year-old man with a history of bladder cancer surgery was admitted in December 2016 for macroscopic hematuria and diagnosed with recurrent malignant bladder cancer complicated by a urinary tract infection. Urine culture yielded >1×10^5^ CFU/mL of a Gram-negative bacillus, identified as *Citrobacter freundii* (WF1033) by MALDI-TOF MS and confirmed by ANI and dDDH analyses.

Antimicrobial susceptibility testing showed that WF1033 was resistant to 18 commonly used antibiotics, including carbapenems, with intermediate susceptibility to cefepime and piperacillin–tazobactam, and susceptibility only to nitrofurantoin and trimethoprim–sulfamethoxazole (**Table 1**). The patient was treated sequentially with cefoxitin and piperacillin–tazobactam and was discharged following clinical improvement.

**Table 1:** Antimicrobial susceptibility testing results of CR-CFR isolate WF1033.

Antimicrobial drug	KB(mm)	MIC(µg/mL)	Antimicrobial susceptibility
Piperacillin-tazobactam		16/4	I
Ceftriaxone		≥64	R
Cefepime		8	I
Cefotetan		≥64	R
Aztreonam		≥64	R
Imipenem		8	R
Ertapenem		≥8	R
Gentamicin		≥16	R
Tobramycin		≥16	R
Amikacin		≥64	R
Ciprofloxacin		≥4	R
Levofloxacin		≥8	R
Trimethoprim-sulfamethoxazole		≤1/19	S
Nitrofurantoin		≤16	S
Ceftazidime		≥64	R
Ampicillin	6		R
Cefoperazone-sulbactam	10		R
Piperacillin	6		R
Cefuroxime	6		R
Meropenem	15		R
Cefoxitin	6		R
Cefazolin	6		R

### Genomic features and carbapenem resistance genes of WF1033

Whole-genome sequencing revealed that WF1033 belongs to sequence type ST116 and consists of a single chromosome (5.00 Mb) and four plasmids (**Table 2**). Three carbapenemase genes, *bla*_KPC-2_, *bla*_IMP-8_, and *bla*_NDM-1_, were identified and distributed across two plasmids.

**Table 2:** Genome features and antimicrobial sensitivity test results of CR-CFR.

Structures	Chromosome	pWF1033-IntI	pWF1033-KPC-IMP	pWF1033-NDM	pWF1033-5
Length (bp)	5004462	236975	57592	53818	14491
GC (%)	51.6	52.2	50.1	49.1	57.7
Total number of ORFs	5114	306	73	52	18
Replicon type	Not applicable	*Inc*FIB(pB171)_1_pB17(accession: AB024946) *Inc*FII(Yp)_1_Yersenia	repA_1_pKPC-2(accession: CP000670)	*Inc*X3_1(accession: CP013325)	Not detected (accession: JN247852)
Antimicrobial resistance gene	*bla*_CMY-135_	*aac*(6’)-Ib-cr, *bla*_OXA-1_, *catB3* *arr*-3, *sul1*, *armA*, *msrE*, *mphE*	*bla*_KPC-2_, *bla*_IMP-8_, *aac(6’)-Ib8*, *bla*_TEM-1_	*bla*_NDM-1_, *bla*_SHV-12_ *ble*_MBL_	Not detected

### Structure of the hybrid plasmid pWF1033-KPC-IMP

**Figure 1:**
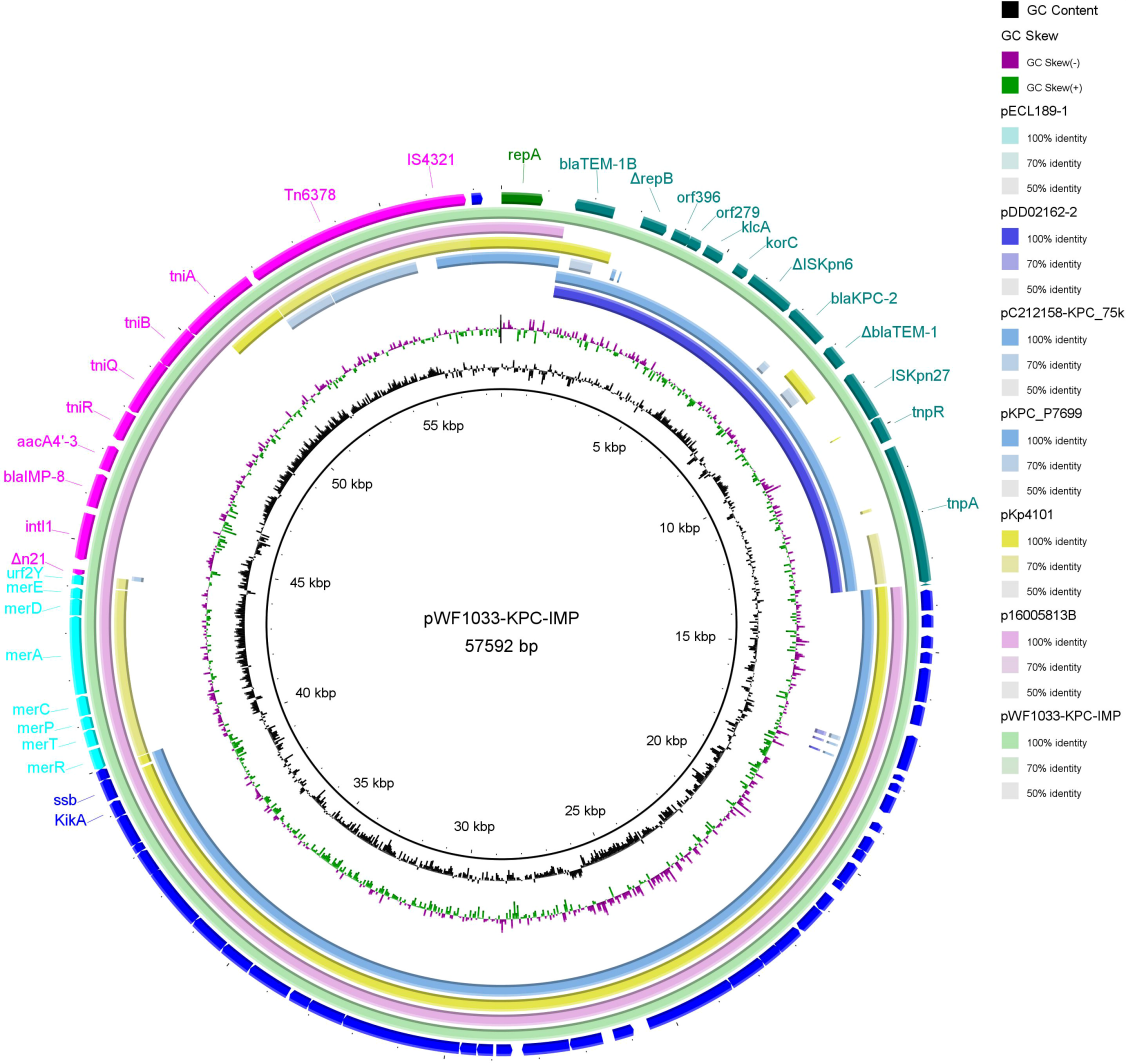
Comparison of pWF1033-KPC-IMP with six closely related plasmids. pWF1033-KPC-IMP was compared with six closely related plasmids, including p16005813B (MK036884.1), pKp4101 (CP047282.1), pKPC_P7699 (CP071913.1), pECL189-1 (CP047966.1), pDD02162-2 (CP087620.1), and pC212158-KPC_75k (CP139398.1), using the BLAST Ring Image Generator.

The novel hybrid plasmid pWF1033-KPC-IMP was found to carry two CRGs: *bla*_KPC-2_ and *bla*_IMP-8_. The regions in which the two CRGs were located were defined as the *bla*_KPC_ region and the *bla*_IMP_ region. Additionally, pWF1033-KPC-IMP was characterized as having a GC content of 50.1% and a total of 73 ORFs, including the ARGs *aac*(6’)-Ib8 and *bla*_TEM-1_ (**Table 2**). Comparative analysis using the NCBI database showed that this plasmid shared the highest nucleotide identity with p16005813B, which carries *bla*_IMP-8_ (GenBank accession no. MK036884.1: coverage, 78%; identity, 99.99%). Notably, the homologous regions were concentrated in areas of the plasmid excluding *bla*_KPC-2_. The novel plasmid also showed high similarity to two plasmids that lack the *bla*_IMP-8_ region: pKp4101 (GenBank accession no. CP047282.1: coverage, 71%; identity, 99.98%) and pKPC_P7699 (GenBank accession no. CP071913.1: coverage, 56%; identity, 99.95%). The *bla*_KPC_ region in pWF1033-KPC-IMP was found to exhibit high similarity to the corresponding regions in pECL189-1 (GenBank accession no. CP047966.1: coverage, 43%; identity, 99.94%), pDD02162-2 (GenBank accession no. CP087620.1: coverage, 21%; identity, 99.78%), and pC212158-KPC_75k (GenBank accession no. CP139398.1: coverage, 21%; identity, 99.78%). Structural comparison of the *bla*_KPC_ region and the *bla*_IMP_ region showed that pWF1033-KPC-IMP was heterozygous for two types of plasmids (**Figure 1**).

**Figure 2:**
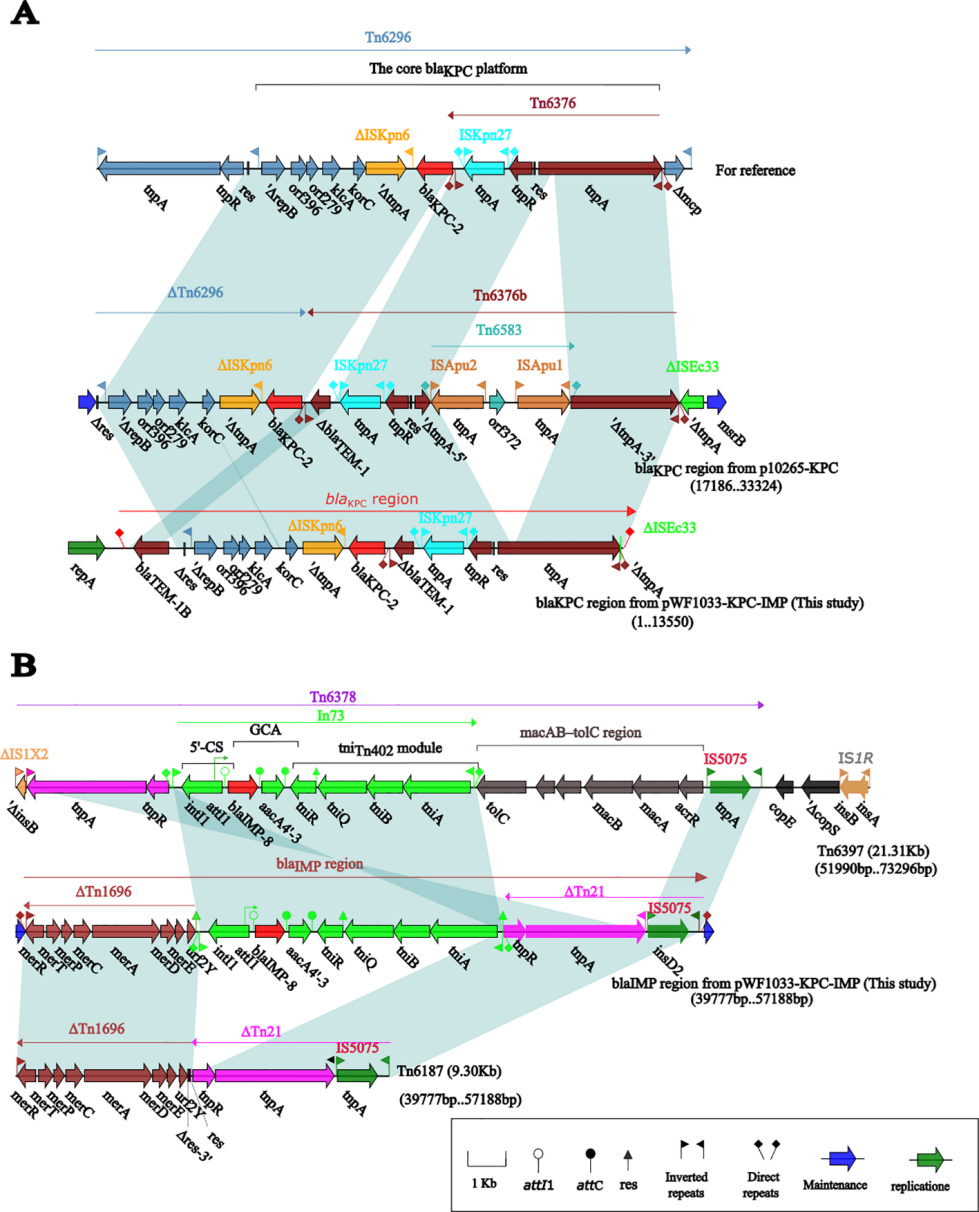
The *bla*_KPC_ region and *bla*_IMP_ region from WF1033 comparison with related regions. **(A)** Organization of the *bla*_KPC_ region from WF1033; **(B)** Organization of the *bla*_IMP_ region from WF1033 Genes are denoted by arrows. Genes, mobile genetic elements, and other features are colored according to their functional classification. Shading denotes regions of homology (light blue: ≥99% nucleotide identity). The accession numbers of p10265-KPC, Tn6397 and Tn6187, which served as references, were KU578314, CP021851, and JQ010984, respectively.

We found that the *bla*_KPC_ region (**Figure 2A**) features direct repeat sequences of TTA spanning nucleotides 1438–13,547 on plasmid pWF1033-KPC-IMP, which are derived from transposon *Tn6376b* and a truncated form of *Tn6296*. This segment features three resistance genes: *bla*_KPC-2_, truncated *bla*_TEM-1_, and intact *bla*_TEM-1b_. Notably, we observed that the *bla*_KPC-2_ gene was embedded within a transposon unit based on *Tn1722*, exhibiting the core structure of *ISKpn*27-*bla*_KPC-2_-Δ*ISKpn*6, similar to that in p10265-KPC. This region differed from *Tn6376b* by the insertion of a truncated *bla*_TEM-1_ gene upstream of *bla*_KPC-2_, but lacked the *Tn6538* insertion. Additionally, an extra *bla*_TEM-1b_ gene component was detected on the left flank of the core *bla*_KPC_ platform, distinguishing it from *Tn6376b*. Overall, the *bla*_KPC-2_ region appears to have evolved from the core *bla*_KPC_ platform through integration of *bla*_TEM-1b_ and *bla*_TEM-1_ between the left flank and *bla*_KPC-2_.

The *bla*_IMP_ region (**Figure 2B**) spans nucleotides 39,971–56,844 of pWF1033-KPC-IMP and features a direct repeat sequence of TATCT. Within this segment, a class I integron integrated the *bla*_IMP-8_ gene, along with the downstream ARG *aacA4*. It shares high similarity with *Tn6378*, particularly in the identical position and sequence of *In73*. However, unlike *Tn6378*, the *bla*_IMP_ region identified in this study was found to lack the *mac*AB*-tol*C region. Additionally, the Δ*IS1*X2-*tnp*A-*tnp*R region located upstream of *In73* is oriented the opposite direction, retaining only the *tnp*R and *tnp*A genes, which together form a truncated *Tn21* with *IS*5075. In addition to the segment from *Tn6378*, the *bla*_IMP_ region identified in this study also shares structural similarity with *Tn6187.* The upstream sequence of *In73* contains Δ*Tn1696*, whereas that downstream retains a truncated *Tn2*. Overall, it appears that *bla*_IMP-8_ integrated into *Tn6187* through *In73* to form this region.

### Structure of the *bla*_NDM-1_–carrying IncX3 plasmid pWF1033-NDM

**Figure 3:**
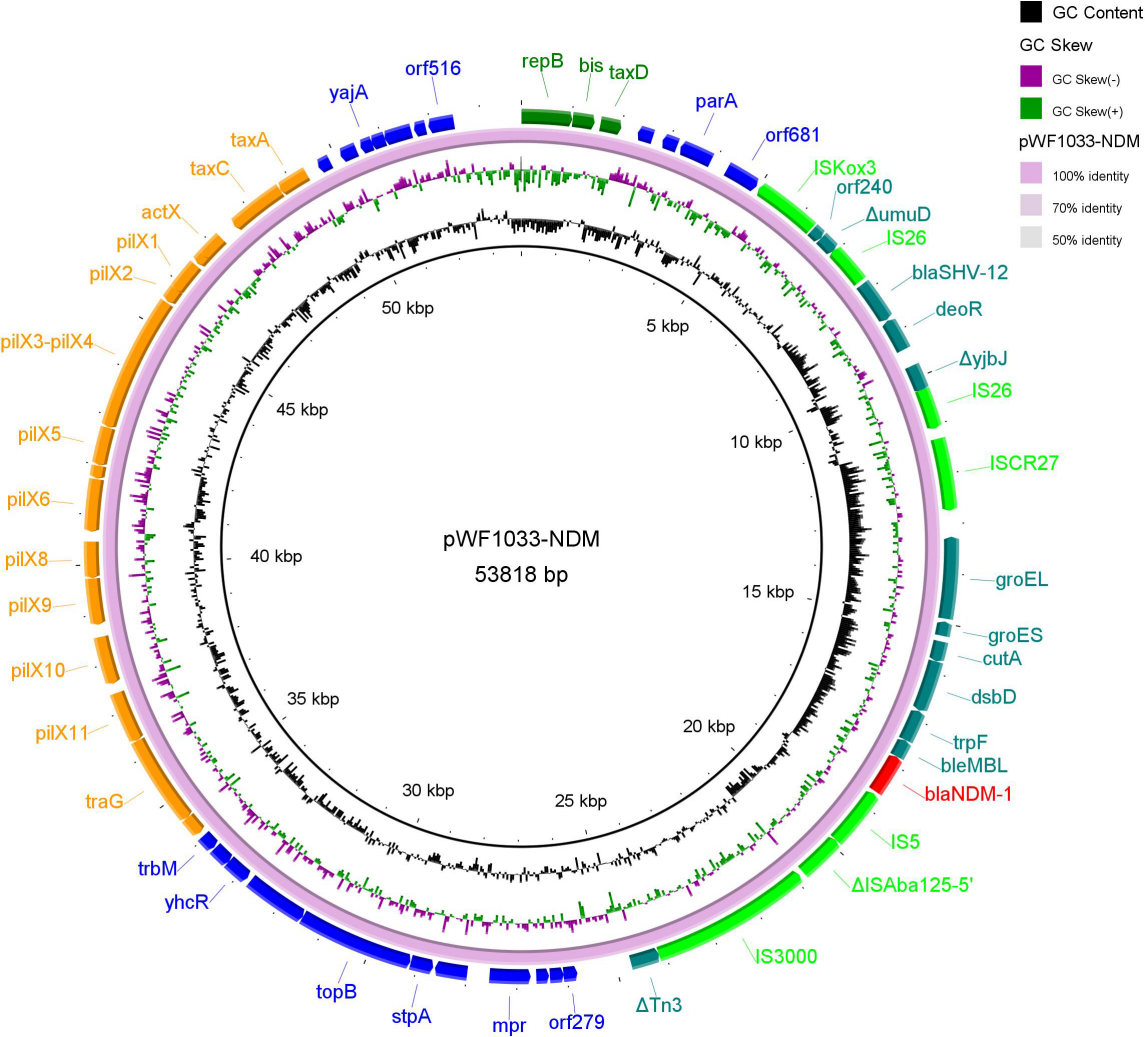
Circular map of the *bla*_NDM-1_-carrying plasmid pWF1033-NDM. Genes are color-coded according to functional categories, and arrows indicate transcriptional orientation.

The plasmid pWF1033-NDM, spanning 53,818 bp with 52 ORFs and a GC content of 49.1%, was identified as an *Inc*X3 type plasmid carrying *bla*_NDM-1_. BLAST analysis against the GenBank database revealed more than 30 known sequences from various carbapenem-resistant bacteria, with 99% coverage and identity. Here, we present only the plasmid structure (**Figure 3**), without comparative circular mapping to other NCBI database sequences.

**Figure 4:**
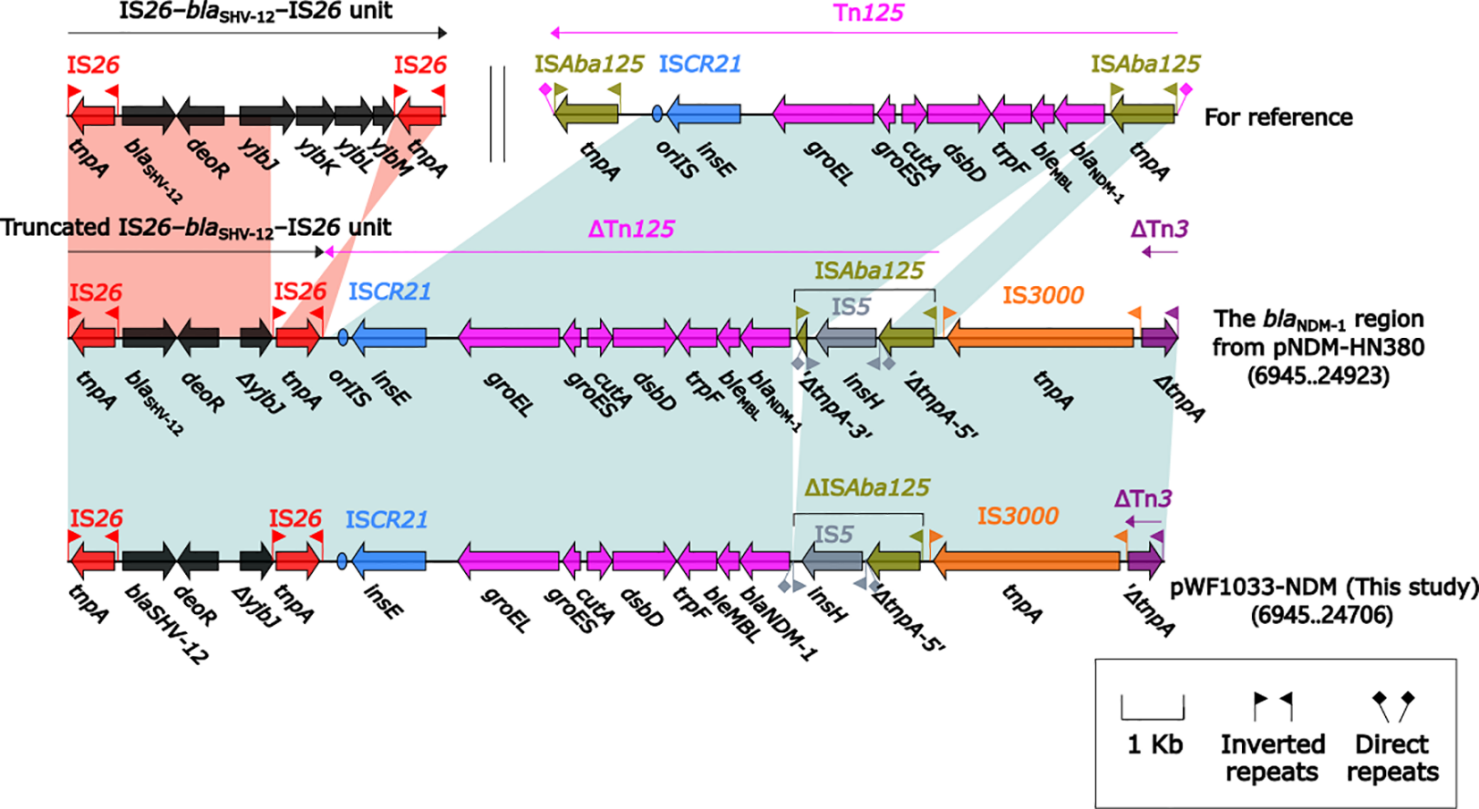
Organization of pWF1033-NDM from WF1033. Genes are denoted by arrows. Genes, mobile genetic elements, and other features are colored according to their functional classification. Shading denotes regions of homology (light blue: ≥99% nucleotide identity). The accession number of pNDM-HN380 used as a reference is NC_019162.

The 54,035 bp *Inc*X3 plasmid pNDM-HN380 (NC_019162) was chosen as the reference sequence for linear alignment. This revealed 99% coverage and >99.9% identity with pWF1033-NDM, which mainly comprises a truncated *IS*26-*bla*_SHV-12_-*IS*26 unit, truncated *Tn125*, *IS*3000, and truncated *Tn3*. The *bla*_NDM-1_ gene is located downstream of Δ*ISAba*125 within the Δ*Tn*125 sequence, which sometimes includes Δ*tnp*A-3′. However, pWF1033-NDM was not found to contain this segment, representing the only difference between pWF1033-NDM and pNDM-HN380 in this study (**Figure 4**).

### The public-genome distribution of carbapenem-resistant *C. freundii*

**Figure 5:**
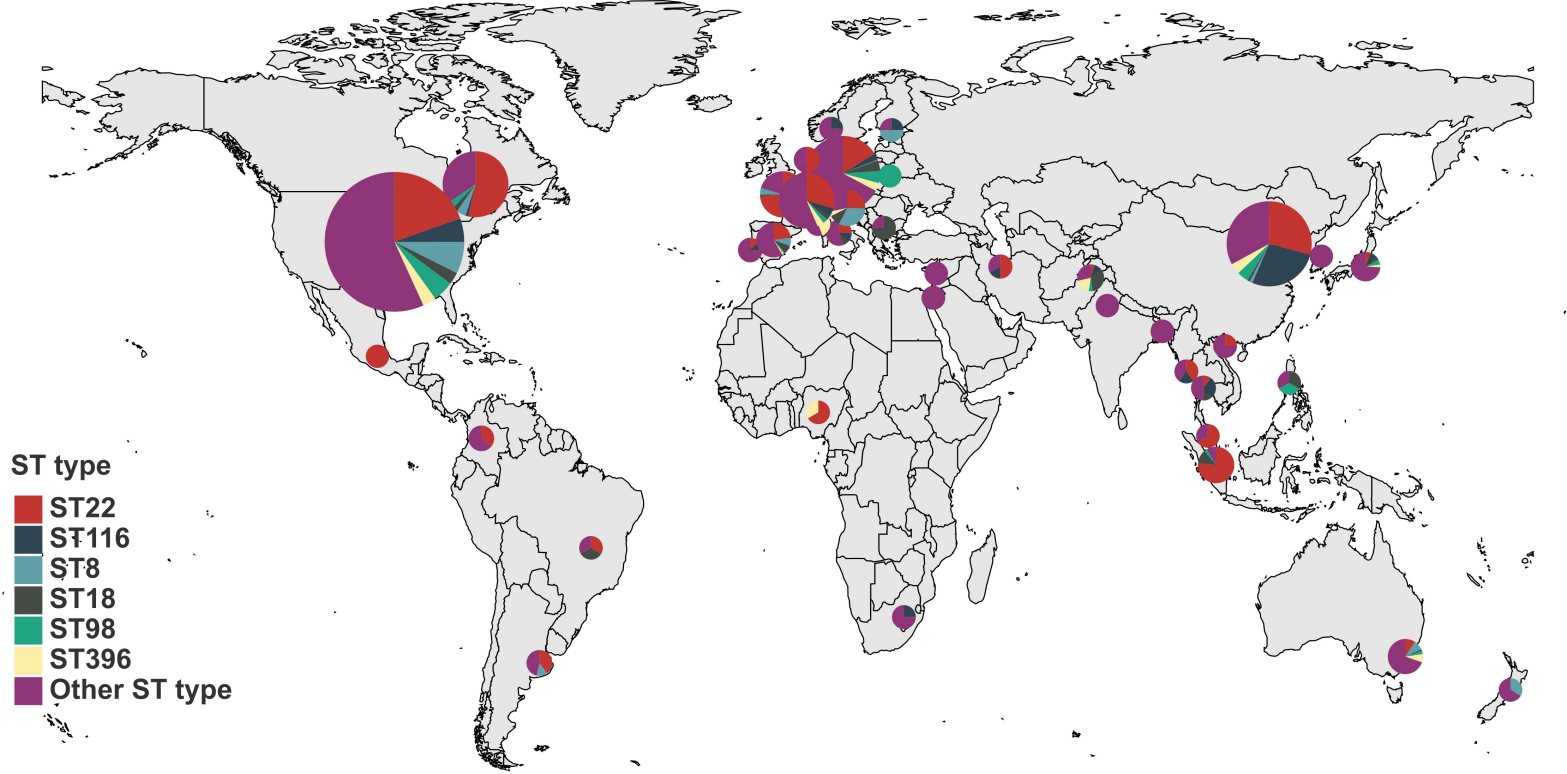
Global map of different ST type of carbapenem-resistant *Citrobacter freundii* in collected strains. Geographical distribution and proportion of different ST types. The different colors in the pie chart indicate different ST types. The size of pies indicates the number of ST types collected in that region.

To analyze the public-genome distribution of CR-CFR, a global CR-CFR distribution map was constructed using data from 2,386 strains (with 50 strains lacking national origin information excluded) (**Figure 5**). Results indicated that CR-CFR is primarily distributed in the United States (641/2,386, 26.87%), China (339/2,386, 14.21%), and Germany (285/2,386, 11.94%).

### Population structure of CR-CFR

**Figure 6:**
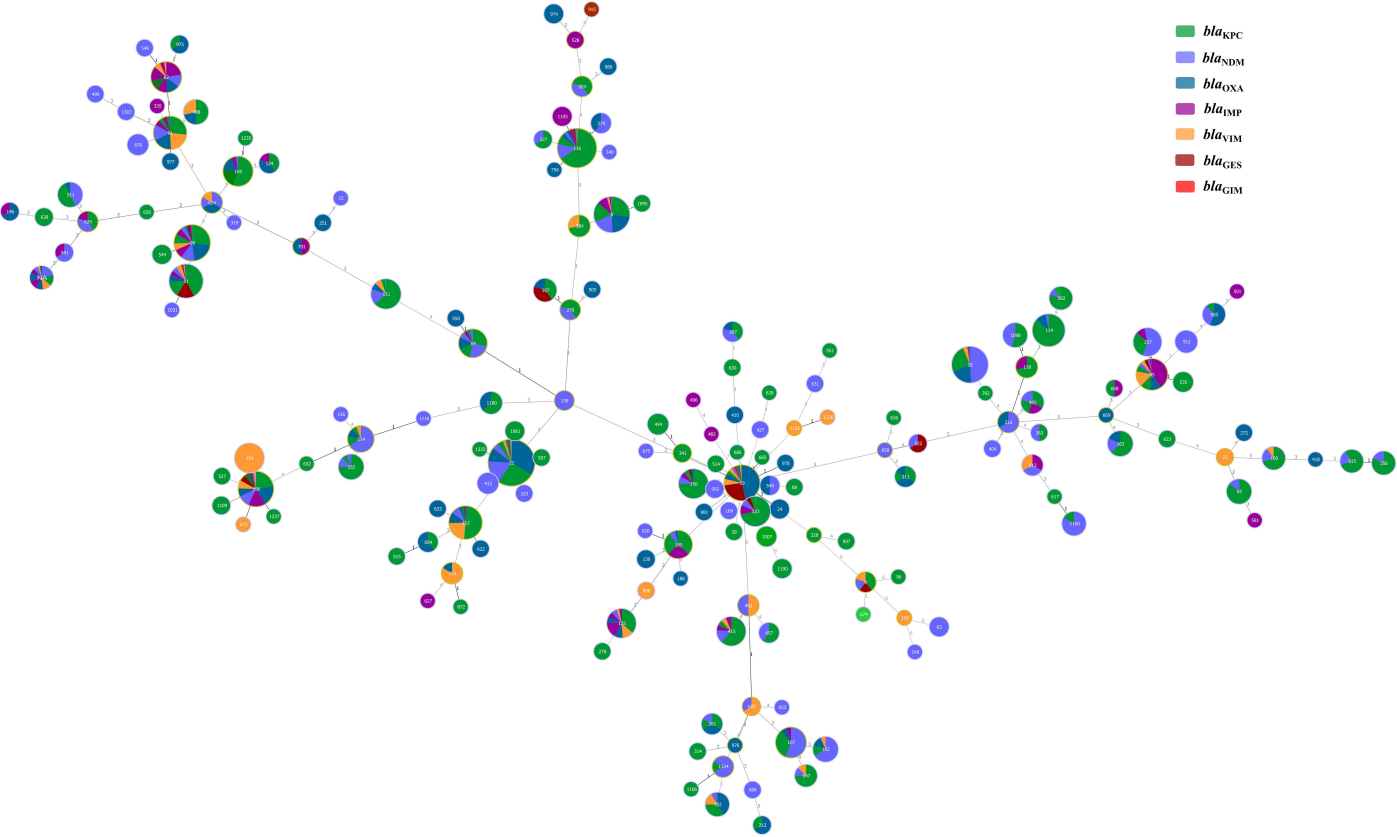
Minimum spanning tree-like structure via the goeBURST full MST algorithm. Each circle represents a type of ST, and different colors represent different types of carbapenem resistance.

To analyze differences in carbapenem resistance gene carriage across various ST types, 2,436 CR-CFR strains were downloaded from NCBI, with a total of 171 ST types predicted (**Supplementary Table 1**). After excluding 94 unclassified ST strains, 2,342 strains were used to construct the minimum spanning tree (**Figure 6**). Among these, ST22 was the most predominant (720/2,342, 30.74%), followed by ST116 (165/2,342, 7.05%), ST8 (108/2,342, 4.61%), ST18 (99/2,342, 4.23%), ST98 (91/2,342, 3.89%), ST396 (71/2,342, 3.03%), and other ST types (1,088/2,342, 46.45%).

Analysis of carbapenem resistance gene carriage in the 2,436 CR-CFR strains revealed that *bla*_KPC_ was the most prevalent (1,093/2,436, 44.87%), followed by *bla*_NDM_ (601/2,436, 24.67%) and *bla*_OXA_ (551/2,436, 22.62%). Among the major ST types, ST22 primarily carries *bla*_OXA_ and *bla*_KPC_; ST116, ST98, and ST8 predominantly carry *bla*_KPC_; ST18 mainly carries *bla*_NDM_; ST95 primarily carries *bla*_IMP_; and ST19 mainly carries *bla*_OXA_.

### Geographic distribution of major ST types

ST22 exhibits the broadest public-genome distribution, with major concentrations in the United States (126/720, 17.50%), Canada (126/720, 17.50%), China (98/720, 13.61%), France (94/720, 13.06%), Singapore (56/720, 7.78%), and the UK (53/720, 7.36%), among other regions.

ST116 has been reported in 16 countries; notably, the majority of isolates (94/165, 56.97%) are distributed in China. While ST22 is the most prevalent ST type in China, ST116 is predominantly distributed in China and ranks as the second most prevalent ST type in the country, trailing only ST22.

The proportions of strains carrying ≥2 CRGs differed among the three groups, with ST116 showing the highest carriage rate (23/165, 13.94%), followed by ST22 (64/720, 8.89%) and other ST types (103/1,551, 6.64%). Results revealed that the carriage rate of ST116 was significantly higher than that of ST22 (χ²=3.87, *P* = 0.049) and also significantly higher than that of other ST types (χ²= 8.98, *P* = 0.003). These results indicate that ST116 differs statistically from other prevalent ST types in the characteristic of carrying multiple CRGs (**Table 3**).

**Table 3:** Comparison of CRGs carriage rates among different STs.

Group	Total	≥2 CRGs	Carriage rate(%)	χ²	*P* value
ST116	165	23	13.94	–	–
ST22	720	64	8.89	3.87	0.049
Other STs	1551	103	6.64	8.98	0.003

Comparisons were performed using the Pearson chi-square test with ST116 as the reference group. *P* < 0.05 was considered statistically significant.

### Phylogenetic analysis of ST116 CR-CFR

**Figure 7:**
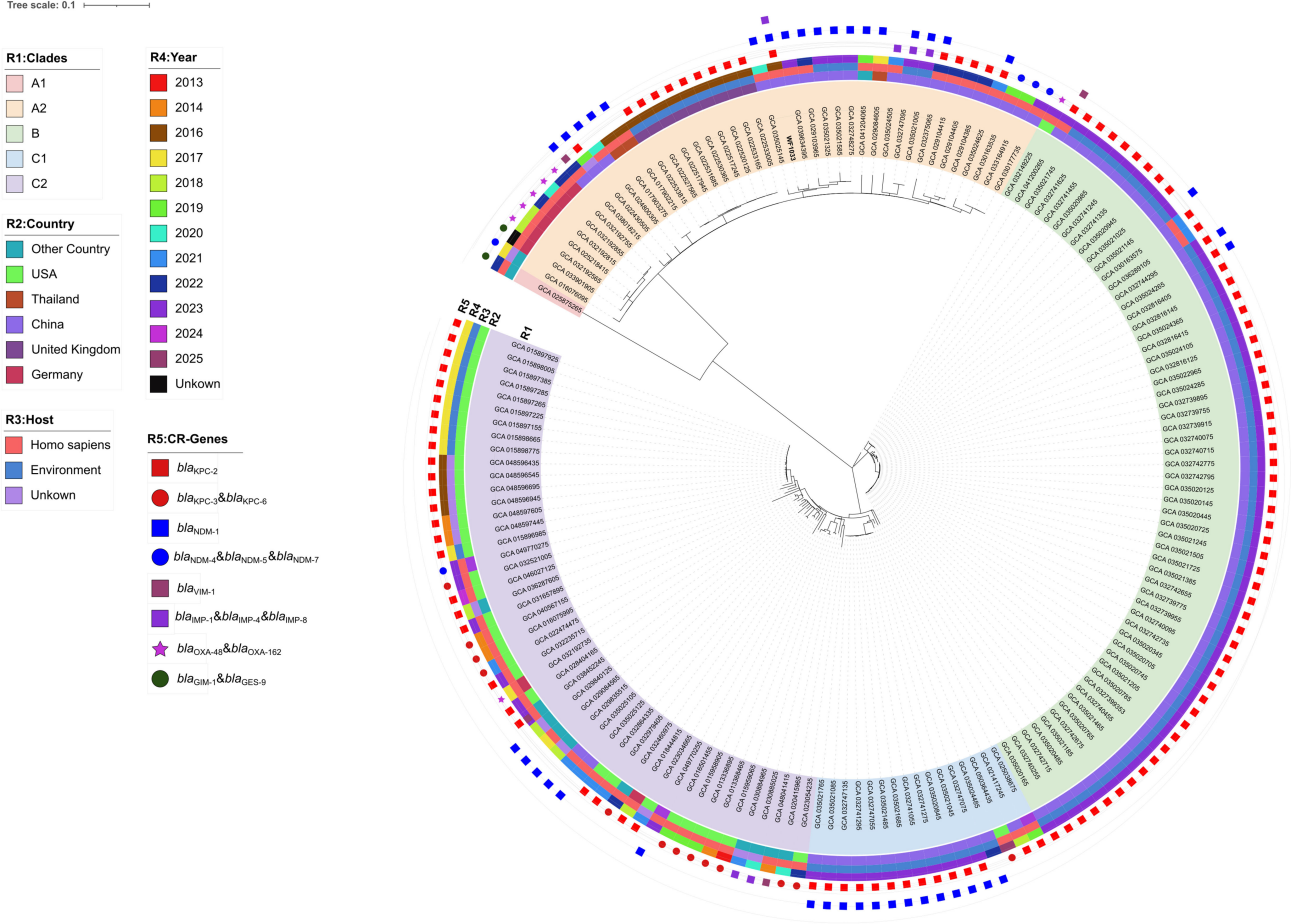
Maximum-likelihood phylogenetic tree of 165 ST116 CR-CFR isolates based on core-genome SNPs. A total of 164 genomes were obtained from NCBI, and WF1033 was included as the study isolate. WF1033 differed by 32 core-genome SNPs from the closest non-redundant human-derived ST116 isolate identified in China.

To analyze the evolutionary relationships and transmission pathways among ST116 strains, a phylogenetic tree was constructed for 165 ST116 CR-CFR strains (**Figure 7**), which were divided into three clades. The experimental strain WF1033 was placed in Clade A2. ST116 strains were most frequently isolated from China (94/165, 56.97%), followed by the United States (35/165, 21.21%). The majority of these strains were derived from environmental specimens (91/165, 55.15%), with the next largest group being of human origin (60/165, 36.36%). A total of 15 carbapenem resistance genes were predicted, with the *bla*_KPC-2_ gene showing the highest carriage rate (112/165, 67.88%), followed by *bla*_NDM-1_ (40/165, 24.24%). A total of 21 strains were found to carry two CRGs simultaneously, and the WF1033 strain in this experiment is the only ST116 strain that carries three CRGs. WF1033 exhibited close phylogenetic relatedness to a human-derived ST116 strain isolated in China in 2023 (GenBank Genome Assembly accession: GCA_039634395), differing by 32 core-genome SNPs.

## Discussion

In this study, we characterized a carbapenem-resistant *C. freundii* isolate exhibiting an extensively drug-resistant phenotype and harboring three distinct carbapenemase genes. Such a resistance profile remains rare in *C. freundii* and underscores the remarkable capacity of this species to accumulate multiple high-level resistance determinants.

Clinically, strain WF1033 was isolated from an elderly patient with recurrent bladder cancer and urinary tract infection, a context consistent with previous reports describing *C. freundii* as an opportunistic pathogen primarily affecting immunocompromised hosts (Wu et al., 2016; Biez et al., 2023; Ye et al., 2023; Zhang et al., 2023a; Aravena-Ramírez et al., 2024). The extensive resistance profile of WF1033, with susceptibility retained to only a limited number of agents, highlights the increasing therapeutic challenges posed by carbapenem-resistant *C. freundii*. These findings reinforce the need for heightened clinical awareness of this species as an emerging reservoir of multidrug resistance in healthcare settings.

A major finding of this study is the identification of a hybrid plasmid, pWF1033-KPC-IMP, co-harboring *bla*_KPC-2_ and *bla*_IMP-8_. Unlike the more commonly reported scenario in which different carbapenemase genes are distributed across multiple plasmids, the coexistence of two carbapenem resistance genes on a single plasmid represents a more compact and potentially advantageous genetic configuration (Din et al., 2023; Yuan et al., 2023; Zhang et al., 2023b; Rodrigues et al., 2024; Zhou et al., 2024). From an evolutionary perspective, such plasmid fusion events may facilitate the co-maintenance of multiple resistance determinants within a single replicon, which could potentially contribute to the persistence and dissemination of multidrug resistance. However, the potential effects on plasmid stability or bacterial fitness require further experimental validation.

Structural analysis revealed that both the *bla*_KPC_ and *bla*_IMP_ regions of pWF1033-KPC-IMP exhibit highly conserved genetic architectures that closely resemble previously reported mobile genetic elements (Wang et al., 2019; Yang et al., 2022; Nobrega et al., 2023; Ye et al., 2023; Zhang et al., 2023a; Zhu et al., 2023; Rodrigues et al., 2024; Zhou et al., 2024). The *bla*_KPC-2_ region is derived from a core *bla*_KPC_ platform with limited structural variation, while the *bla*_IMP-8_ region is embedded within a class I integron framework closely related to In73. The high degree of conservation observed in these regions suggests that carbapenemase genes are frequently mobilized as stable genetic modules rather than through extensive *de novo* rearrangements. Such modularity likely facilitates repeated integration into diverse plasmid backbones, contributing to the emergence of hybrid plasmids carrying multiple resistance determinants.

In addition to plasmid fusion, WF1033 carries *bla*_NDM-1_ on a typical IncX3 plasmid, a plasmid family well recognized for its high conjugation efficiency and broad host range (Sánchez-Urtaza et al., 2023; Verma et al., 2023; Di Marcantonio et al., 2024; Rezzoug et al., 2024; Rodrigues et al., 2024; Zhou et al., 2024). IncX3 plasmids have been widely implicated in the global dissemination of *bla*_NDM_ variants across multiple Enterobacterales species (Biez et al., 2022; Zhang et al., 2023a; Aravena-Ramírez et al., 2024). The coexistence of an IncX3-*bla*_NDM-1_ plasmid with a hybrid *bla*_KPC_*/bla*_IMP_ plasmid within a single host further illustrates the exceptional capacity of *C. freundii* to act as a convergence point for multiple high-risk resistance elements.

Beyond plasmid-level mechanisms, our large-scale genomic analysis identified ST116 as a lineage strongly associated with the accumulation of carbapenem resistance genes in CR-CFR. Although ST22 remains the most prevalent sequence type globally (Hadjadj et al., 2022; Hamerlinck et al., 2023), ST116 shows pronounced regional enrichment in China (Ju et al., 2025; Zhao et al., 2025) and a significantly higher propensity to carry multiple carbapenem resistance genes. Notably, ST116 strains exhibited the highest proportion of isolates harboring two or more carbapenemase genes, and WF1033 was the only ST116 strain identified to carry three distinct carbapenemase genes. These findings suggest that ST116 is not merely a frequent clone, but rather a genetic background potentially associated with the accumulation and co-occurrence of multiple resistance determinants.

Phylogenetic analysis further indicates that ST116 strains are widely distributed across environmental and clinical sources, with environmental isolates accounting for a substantial proportion of the population. This observation suggests that environmental reservoirs may contribute to the maintenance and circulation of ST116-associated resistance plasmids and CRGs, potentially facilitating transmission into clinical settings. Pairwise SNP analysis demonstrated that WF1033 differed by 32 core-genome SNPs from the closest non-redundant human-derived ST116 isolate identified in China, supporting their close phylogenetic relatedness and possible regional circulation rather than confirmed transmission events.

Taken together, our findings support a model in which the convergence of a permissive genetic background associated with ST116 and highly adaptable plasmid architectures enables the stepwise accumulation and co-occurrence of multiple carbapenemase genes in *C. freundii*. This evolutionary trajectory is particularly concerning, as it combines efficient horizontal gene transfer with lineage persistence across environmental and clinical niches. Continuous genomic surveillance, with particular attention to ST116 and hybrid resistance plasmids, is therefore essential to mitigate the further spread of extensively drug-resistant *C. freundii* and to inform effective infection control strategies.

## Conclusion

In conclusion, this study highlights *C. freundii* as an underrecognized but highly adaptable host capable of integrating diverse carbapenem resistance determinants through plasmid-mediated recombination. Our findings suggest that the interaction between clonal background and mobile genetic elements plays a central role in enabling the persistence and dissemination of extensively drug-resistant *C. freundii*. The emergence of such multidrug-resistant configurations in both clinical and environmental contexts underscores the importance of continued genomic monitoring to support early detection and effective infection control interventions.

## Data Availability

The datasets presented in this study can be found in online repositories. The names of the repository/repositories and accession number(s) can be found in the article/**Supplementary Material**.
